# Comparison of Maximum Heat Generation during Implant Site Preparation between Single and Gradual Drilling Protocols in Artificial D1 Bone Blocks: An In Vitro Study

**DOI:** 10.1155/2022/9370395

**Published:** 2022-06-18

**Authors:** Tammam koutiech, Omar Ahmad heshmeh, Kamal Alkerdi, Johnny Toumi, Laith Al sabek

**Affiliations:** ^1^Damascus University Faculty of Dentistry, Department of Oral and Maxillofacial Surgery, Damascus, Syria; ^2^Department of Physics and Laser Technology, High Institute for Laser Research and Applications, Damascus University, Damascus, Syria; ^3^Faculty of Dentistry, Damascus University, Al-Mazzeh St., Damascus 30621, Syria

## Abstract

**Aim:**

Two protocols for implant site preparation have been demonstrated in the literature: conventional gradual drilling and single drilling.

**Objectives:**

The purpose of this study is to assess the maximum temperature changes reached during and after implant site preparation of 4.2 implant diameter using the gradual drilling protocol and single drilling protocol. *Material and Methods*. The artificial bone block samples (#1522–23; Sawbones, Malmö, Sweden) with the density of d1 were divided into two groups. Twelve implant site preparations were performed with the use of only one drill in group A, and the same number of preparations was performed using five gradual drills in group B. The drilling speed was set for each group at 1500 rpm with the use of external irrigation with a constant of 50 ml/min at room temperature (25 ± 1°C). The maximum temperature changes were measured using an infrared camera (Fluke Ti55, USA). The data were gathered and analyzed using Student's *t*-test for independent samples.

**Results:**

With the 95% confidence intervals (CIs) for the means of ∆T between groups A and B, group B showed a statistically significant higher temperature change (∆T) than group A.

**Conclusions:**

The current outcomes propose that the single drilling protocol, while preparing a bed for a 4.25 mm dental implant in d1 artificial bone blocks, generates less heat than the conventional gradual drilling protocol.

## 1. Introduction

Due to their 10-year success rate of 97%, endosseous dental implants have become an increasingly favored method of therapy in dentistry [1].

Many studies in the literature have shown that healing of the bone after osteotomy preparation for implant placement is essential to the onset of an acceptable osseointegration and the long-term success of dental implants [2, 3].

Authors considered a direct, atraumatic, delicate, surgical procedure to be the determining factor for the implant's success because thermal or mechanical harm caused during the preparation of the implant site could trigger bone necrosis and failure of the osseointegration process [4, 5].

Heat generation during drilling of the bone in order to receive the implant has been recognized as a risk factor, whereas excessive drilling temperatures and continuation of these high temperatures can cause complications such as delayed healing as well as necrosis [6].

Previous studies, both in vivo and in vitro, have focused on the factors that affect and compromise the osseointegration process and cause an increase in the heat generated during the implant site preparation. Among these factors are drilling turning speed, load applied during osteotomy, osteotomy time, sharpness, manufacturing materials, general condition of the drills, irrigation and irrigation system, drill geometry, surgical techniques used, and bone structure [7].

Other studies used computational models and finite element methods to simulate the human bone in terms of density and thermomechanical characteristics, drilling instrumentation, and experimental conditions in order to have a better control on the parameter and to analyze the drilling procedure and its thermal effect on the bone [8, 9].

The description of different drilling techniques and the heat generation during and after drilling in these different techniques have both been subject to previous studies and research studies [10].

Osteotomy preparation can be achieved by drilling with one single drill or conventional gradual drills [11].

The conventional gradual method depends on preparing the implant site by using a set of drills that increases in diameter to remove small quantities of the bone. This method is based on the hypothesis that says conventional drilling decreases the heat generation caused by drilling, whereas this assumption relates the amount of heat generation with the amount of the bone removed by each drill [12–15].

However, other studies showed that heat generation was related to the duration of the drilling and friction forces applied to the bone while preparing the implant site [4, 16].

Only few studies have compared the single and gradual osteotomy preparation techniques to demonstrate the optimal technique concerning heat generation in the bone [17].

However, no optimal surgical drilling technique has been described.

Different bone samples and temperature measurement techniques were included in the previous studies that investigated the heat generated during implant site preparation.

The main in vitro methods for measuring the temperature during osteotomy preparation are as follows: a thermocouple with probes that can be inserted inside the sample for recording the temperature, an infrared camera provides an indirect measurement and thermal readings for the preparation site, and an infrared thermometer has been used in some research studies [7, 9, 18].

Furthermore, bone sample types have varied. In vitro studies have usually used bovine bone ribs, synthetic bone models (polyurethane foam), or human ex vivo bone [19–23].

The advantage of the polyurethane bone foams is that they allow valid reproducibility which leads to less susceptibility to errors [18].

However, in terms of bone density, a little attention was given to the bone samples that were used in previous studies, since most of these studies have used bovine bone samples that, according to Misch classification, are d3 or d4 in terms of their density [24–26]. Whereas, in the human maxilla and mandible, different bone densities and structures (between D1 and D4) can be found, which call for questioning or reconsideration in terms of these studies' designing, especially since artificial bone blocks with different densities are available.

Thus, the aim of this study was to evaluate the maximum temperature generated in two different drilling protocols to prepare a bed of 4.25 mm implant on d1 polyurethane artificial bone blocks.

## 2. Materials and Methods

### 2.1. Sample Size Determination

Sample size was calculated using the G-power 3.1 software. The significance level was set at 0.05 and the power of the study was set to be 95%. The sample size was determined to be 12 sites for each group.

**Figure 1:**
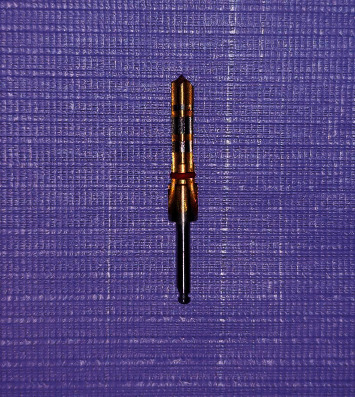
Single drill for preparing the final implant bed for 4.25Ø mm implant; the single drill design contains 3 straight flutes with wide channels between them (FGM, Arcsys).

**Figure 2:**
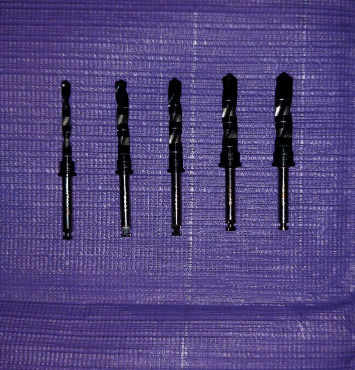
Gradual implant drills for the final implant bed for 4.25Ø mm implant; the gradual drill designs contain twisted flutes and only the pilot drill have a working tip (GMI, Frontier).

The sample was divided into two groups (12 sites per group):

  Group A: 12 implant bed preparation for 4.25 mm implant diameter were prepared in the single drill system with a 4.0 mm drill (Arcsys, FGM) (Figure 1)
  Group B: 12 implant sites were prepared for 4.25 implant diameter in the gradual implant system using 5 drills (2,0/2,8/3.0/3.5/4.0) (Frontier, GMI) (Figure 2)

### 2.2. Research Protocol

**Figure 3:**
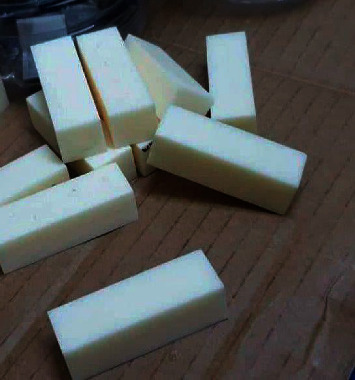
D1 artificial bone blocks made from solid rigid polyurethane foam (SRPF) (#1522–23; Sawbones, Malmö, Sweden).

The implant site osteotomy was performed on d1 artificial bone blocks (#1522–23; Sawbones, Malmö, Sweden). The solid rigid polyurethane foam (SRPF) blocks used in this study are classified according to density d1, 0.48 g/cm^3^ (Figure 3).

We set a drilling depth of 12 mm for the two groups. The drilling speed was approximately 1500 rpm, and external irrigation was achieved with a constant 50 ml/min at room temperature (25 ± 1°C) and a new set of drills used for each group.

**Figure 4:**
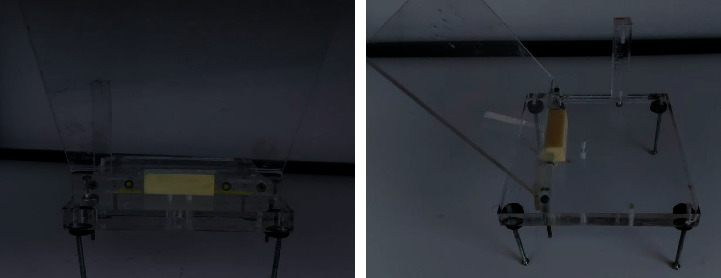
The design of the bracket. The special bracket was designed for holding the samples during the osteotomies, and a gingival protector was used also to isolate the surface where the temperature was measured in order to prevent the irrigation from interfering with the infrared camera readings and giving rise to measurement errors.

For this experiment, we constructed a special bracket for holding the samples during the osteotomies and for isolating the surface where the temperature was measured in order to prevent the irrigation from interfering with the infrared camera readings and giving rise to measurement errors (Figure 4).

The initial temperature of the artificial bone samples and the drilling kits was similar to room temperature (in the range of 22.0–28.7°C).

Each drill was positioned in such a way on the polyurethane foam blocks that after drilling the preparation site, the thickness of the remaining wall was between 0.3 mm and 0.7 mm in order to locate the osteotomy in the area where the infrared camera can estimate the temperature variations. This design was chosen in order to reduce bias influencing the temperature measures.

We placed the infrared camera 50 cm away from the test samples for maximum spatial resolution.

**Figure 5:**
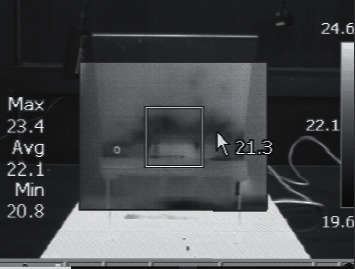
Thermal videos taken during drilling on the bone samples.

Thermal videos of the drilled blocks were taken during and after the drilling using a digital infrared camera (Fluke Ti55, USA) (Figure 5).

The infrared camera was set by the following parameters: the distance between the infrared camera and the bone sample was 50 cm, the emissivity was set at 95%, the room temperature was 25 ± 3°, and the relative humidity was 50%.

### 2.3. Statistical Analysis

In this study, the level of significance (*p* value) and power of the study were set at 0.05% and 90%, respectively. Student's *t*-test for independent samples was used with the 95% confidence intervals (CIs) for the means of ∆T between groups A and B.

The IBM SPSS software v. 23 was used to perform the statistical analysis.

## 3. Results

The sample consisted of 24 implant site preparations, which were divided into two main groups (A and B), two equals, according to the method of preparing the implant site (using the single drilling protocol or the conventional drilling protocol), and the sample was distributed according to the method of preparing the implant site as given in Table 1.

The initial temperature (T0) and the maximum temperature (*T*_max_) in both groups were measured and recorded, as given in Table 2.

The maximum change in temperature ∆*T*= (*T*_max_–T0) for the groups A and B is given in Table 3.

The mean, standard deviation, standard error, and maximum and minimum limits of ∆T are given in Table 4.

Student's *t*-test for independent samples was used with the 95% confidence intervals (CIs) for finding the means of ∆T between groups A and B, as given in Table 5.

The previous tables show that the maximum change in temperature ∆T in group A was less in comparison with group B with a statistically significant difference (*P* value = 0.004), which means that the heat generated in the single drilling protocol was less than the conventional gradual drilling protocol when preparing for a 4.25Ø implant.

## 4. Discussion

This experiment's intention was to investigate the differences in the temperatures generated during drilling in the single drill protocol and the conventional gradual drilling protocol.

To measure the changes in the temperatures during drilling, we used a thermal infrared camera (Fluke Ti55, USA). The reason that we used the infrared camera was that it has several advantages over the thermocouple such as it measures the overall thermal profile, while the thermocouple measures only the spot temperatures near the probe and also the thermal camera records the temperatures without any contact with the bone surface [10, 19, 27].

However, it is important to clarify that the disadvantage of the thermal camera is the inability to record the real temperatures through liquids, and therefore, it cannot measure the temperature on the bone surface wetted in serum, so in order to solve this problem, we designed a special appliance to hold and isolate the bone sample surface from the irrigation pumped while drilling (Figure 4).

We used in this experiment d1 artificial bone blocks #1522–23; Sawbones, Malmö, Sweden) that have been tested in other dental implant studies [18, 28].

These blocks are considered to be standardized and accepted by the ASTM (American Society for Testing and Materials) for testing orthopedic devices and instruments, which makes them ideal for the comparative testing of different drilling protocols for dental implants.

The solid rigid polyurethane foam (SRPF) blocks used in this study are classified according to density d1, 0.48 g/cm^3^. The reason for using artificial bone blocks is because of good reproducibility due to less sensitivity to errors; in addition, the artificially manufactured bone is reported to provide equal vertical and horizontal parameters, allowing for standardized bone density. Other studies used bovine ribs as a drilling sample; however, the disadvantages for using bovine ribs are the variation in density and grade of mineralization and also the fluid retained on the bone surface that could jeopardize the accurate temperature reading. New studies have used human ex vivo bone samples; however, the main disadvantages of these samples are the lack of standardization and reproducibility due to the variation in both the density and the shape of the bone samples [10, 23].

We used in this study artificial bone block samples with a density of d1, since the d1 bone density is considered to be the most vulnerable to overheating among the four types of bones [28].

After all the preparatory steps of this experiment were completed, the initial temperature T0 was considerably varied (in the range of 22.0–28.7˚C), so we chose the maximum change in the temperature ∆T caused by the drilling procedures. This decision was taken into consideration of thermodynamics' first law, which states that the variety in the inward energy of a thermodynamic system corresponds to the amount of heat and work entering the system, so the quantity of heat absorbed by the sample and its consequent increase in temperature are independent of the initial temperature T0 of the sample [11].

The thickness of the remaining wall between the preparation site and the surface where the temperature was measured was between 0.3 mm and 0.7 mm, so that the infrared camera could read the changes in temperature accurately, whereas Matthews and Hirsch published that the temperature measured on the surface of the bone sample decreases proportionally to the increase in the distance between the drilling site and the surface where the temperature was measured [29].

In our experiment, we drilled the bone samples at a turning speed of 1500 rpm, which was recommended by Eriksson and Adell (1986) [17], whereas earlier studies claimed that a low turning speed while drilling generates less heat [7, 30].

However, recent studies have shown that lower turning speeds are associated with low cutting efficiency and more heat generation during drilling [31].

Furthermore, the turning speed of the drill itself cannot be considered as an independent factor in heat generation during drilling without associating it with the load applied during drilling [32].

The load applied during drilling was left to the experienced operator's hand, leaving him free to adjust the load applied according to the resistance while the drill is moving forward in the bone, since according to Misch, the pressure applied during drilling should be neither heavy enough to obstruct the turning of the drill (which affects the cutting efficiency and increases the heat generated) nor light enough to result in heat generation without removing the bone [31].

We irrigated the drilling procedure with external irrigation using saline solution at room temperature with a constant pumping of 50 ml/min. We chose the irrigation system and the amount of irrigation based on Sener et al. and Rashed et al.' studies that concluded that room temperature solution provides sufficient cooling during drilling and that a higher volume of irrigation was not associated with decreasing the heat generated from drilling [3, 16].

The variation in the temperature readings in spite of using artificial uniformed d1 bone blocks might be because of the natural tolerance of the amount, speed, and accessibility of the cooling irrigation during drilling.

### 4.1. Limitation of the Study

The study conditions for this experiment differ from those common in in vivo studies, as we applied the osteotomy protocols on artificial bone blocks that do not have blood flow and do not have the same body temperature as the vital human bone. Therefore, the temperatures change during drilling in this study can be presumed to be unidentical to the changes in temperatures in an in vivo study on the human bone. Another limitation of this study was the inability to evaluate the microcracks that could happen during implant site preparation, and also, we only studied the heat generation using only d1 bone blocks.

## 5. Conclusion

Within the limitations of the study, the current outcomes propose that a single drilling protocol while preparing a bed for a 4.25 mm dental implant in d1 artificial bone blocks generates less heat than the conventional gradual drilling protocol and that could be attributed to the differences between the drill shape and design of the two drilling systems; the single drill burs design that consist of 3 straight flutes allow Weider channels between these flutes that enable the elimination of cutting debris and reduce the drill-bone contact area which leads to reduce the frictional resistance and the heat generated, while the twisted shape of the gradual drills could narrow these channels between the cutting edges of the drill, and the nonworking tip design of the gradual drills (except the 2 mm pilot drill) increases the drill-bone contact area and the heat generated.

These results support the hypothesis that a single drilling protocol is not only applicable in implant site preparation without overheating the bone but also reduces the heat generated on the bone. However, further research has to be done in order to assess the microfractures that could happen during single drilling and to describe the optimal site preparation technique to lessen the damage to the bone during osteotomy. Also, more clinical studies should be done to evaluate the implant success rate while using the single drill technique.

### 5.1. Clinical Relevance

From the clinical point of view, these two techniques have several advantages and disadvantages: the gradual drilling technique allows the surgeon to modify the axis of the implant after using the first drill (pilot drill), but it will a take longer period of time to prepare the final implant bed, which led to more discomfort to the patient and prolong the surgical procedure especially when multiple implants are to be placed, and may cause a bigger inflammatory response and more after surgery pain for the patient, whereas the single drilling technique reduces the time needed to prepare the final implant bed and reduces the duration of the surgical intervention which makes it more acceptable by both clinicians and patients; however, axis modification of the implant bed is not possible in this technique, which inquires greater precision from the surgeon or the use of a computed surgical guide [33], [34, 35].

## Figures and Tables

**Table 1:** The sample distribution according to the method of implant site preparation.

Percentage	Number of implants site preparations	Drilling protocol
50.0	12	Single drilling protocol
50.0	12	Conventional drilling protocol
100	24	Total

**Table 2:** The initial temperature T_0_ and the maximum temperature *T*_max_ recorded during implant site preparation.

Drills Ø	Conventional drills										Single drill	
	2.0 mm		2.8 mm		3.0 mm		3.5 mm		4.0 mm		4.0 mm	
	T_0_	*T*_max_	T_0_	*T*_max_	T_0_	*T*_max_	T_0_	*T*_max_	T_0_	*T*_max_	T_0_	*T*_max_
1	23.6°	26.9°	23.3°	34.4°	25.0	29.4°	24.0°	24.8°	23.5°	24.2°	22.3°	30.2°
2	23.3°	26.1°	23.0°	28.3°	25.0	31.4°	23.0°	24.8°	23.5°	26.6°	22.0°	26.2°
3	24.6°	29.4°	25.0°	34.3°	23.5°	24.6°	24.0°	25.8°	24.5°	25.6°	23.0°	24.8°
4	25.5°	28.1°	23.5°	31.3°	23.5°	24.2°	24.5°	26.2°	24.5°	27.3°	22.0°	29.9°
5	25.5°	27.9°	25.0°	29.0°	25.5°	28.7°	24.5°	25.9°	24.5°	26.1°	23.0°	27.7°
6	25.5°	32.1°	25.0°	38.1°	25.5°	29.0°	25.0°	26.1°	24.5°	27.2°	22.5°	34.1°
7	28.5°	33.2°	26.0°	36.0°	26.3°	32.3°	25.0°	28.5°	24.5°	25.9°	22.5°	24.0°
8	28.5°	32.3°	26.0°	30.8°	26.3°	29.4°	26.2°	34.0°	25.0°	26.9°	22.5°	33.0°
9	27.3°	30.0°	26.3°	45.0°	26.3°	28.8°	26.2°	34.1°	25.0°	26.2°	28.5°	31.5°
10	27.3°	30.0°	26.3°	34.3°	26.3°	30.4°	27.4°	36.3°	27.0°	36.3°	28.5°	30.2°
11	27.0°	32.8°	26.3°	37.2°	26.3°	31.7°	28.7°	33.8°	27.0°	32.8°	28.2°	31.3°
12	26.3°	29.3°	26.0°	36.5°	26.0°	31.8°	28.0°	32.8°	27.0°	33.1°	28.2°	32.3°

**Table 3:** The maximum change in temperature ∆*T*= (*T*_max_–T0) for the groups A and B.

Drills Ø	Conventional drills					Single drill
	2 mm	2.8 mm	3 mm	3.5 mm	4 mm	4 mm
	∆T					∆T
1	3.3°	1.1°	4.4°	0.8°	0.7°	7.9°
2	2.8°	5.3°	6.4°	1.8°	3.1°	4.2°
3	5°	9.3°	1.1°	1.8°	1.1°	1.8°
4	2.6°	7.8°	0.7°	1.7°	2.8°	7.9°
5	2.4°	4°	3.2°	1.4°	1.6°	4.7°
6	6.6°	3.1°	3.5°	1.1°	2.7°	11.6°
7	4.7°	10°	6°	3.5°	1.4°	1.5°
8	3.8°	4.8°	3.1°	7.8°	1.9°	10.5°
9	2.7°	19°	2.5°	7.9°	1.2°	3°
10	2.7°	8°	4.1°	8.9°	9.3°	1.7°
11	5.8°	10.9°	5.4°	5.1°	5.8°	3.1°
12	3°	10.5°	5.8°	4.8°	6.1°	4.1°

**Table 4:** The mean, standard deviation, standard error, and maximum and minimum limits of ∆T.

Preparation method	*N*	Mean	SD	Se	∆*T*_max_ limit	∆*T*_min_ limit
Group A	12	5.17	3.47	1.00	1.5	11.6
Group B	12	9.91	3.66	1.06	4	18.7

**Table 5:** Confidence intervals (CIs) for the means of ∆T between groups A and B.

The study variant	Mean difference	T	Sig. (2-tailed)
∆T	−4.74	−3.256	0.004^*∗*^

^*∗*^Statistically significant difference.

## Data Availability

The data used to support the findings of this study are available from the corresponding author upon request.
